# Expression of the metalloproteases MMP-1, MMP-2, MMP-3, MMP-9, MMP-11, TIMP-1 and TIMP-2 in angiocentric midfacial lymphomas

**DOI:** 10.1186/1477-7819-6-114

**Published:** 2008-10-27

**Authors:** Abelardo Meneses-García, Alejandro Mohar Betancourt, Jorge Herrera Abarca, Adriana Becerril Montes, Lourdes Suarez Roa, Luz Ruíz-Godoy

**Affiliations:** 1 Pathology department, Instituto Nacional de Cancerología, México; 2 Pharmacology department, Instituto Politécnico Nacional, México; 3 Basic research, Instituto Nacional de Cancerología, México

## Abstract

**Background:**

Extranodal T/NK cell lymphomas possess distinctive clinico-pathological characteristics: they are angiocentric, exhibit extensive necrosis. Prognosis is poor in the short term. The objective is to explore the expression of different MMPs in the cells and stroma which are around of the blood vessels damaged and their correlation with clinico-pathological parameters.

**Patients and methods:**

Twenty cases of this type of lymphomas were studied and collected patient clinical data. The expressions of MMP-1, 2, 3, 9, 11, 13 and TIMP-1, 2 were studied by immunohistochemistry. Ultrastructural studies were performed in two cases. Statistical analysis was done with Fisher's exact test, Chi^2 ^test.

**Results:**

Of the 20 patients, 13 were men with median age of 43 years. In 13 patients the primary tumor was localized in the nasal cavity. Treatment was combined chemotherapy and radiotherapy in 60%. The 55% advanced clinical stages, 70% died from the disease. There were neoplastic cell and peritumoral fibroblasts positivity to MMP-1 and MMP-11 in most of the cases. The MMPs-2, 3 and 9 were expressed in neoplastic cell between 30 to 65%of the cases. TIMP-1 was presented mainly in the epithelium and TIMP-2 was poor expressed of the all cases.

**Conclusion:**

There were no statistical significance between the different enzymes used and the clinical parameters, besides status and survival of the patients. It is necessary to study more enzymes and focus them to quantify and determine their activity, in order to have a better correlation with histological features in this type of neoplasm.

## Background

Nasal-type extranodal T/natural killer (T/NK) cell lymphomas represent a distinctive clinico-pathological entity, characterized by progressive destruction. This type of lymphoma generally originates in the nasal cavity, the palate or midfacial region, and its main characteristic is angioinvasion and angiodestruction, accompanied by extensive areas of necrosis [1-4].

The typical localization of this neoplasm is the nasal cavity; however, it may also appear in the palate, adjacent anatomic regions and/or distant tissues, such as gastrointestinal, testicular, liver, spleen, central nervous system, and even lymph node tissue [1,5,6]. Nasal type extraganglionary T/NK cell lymphomas have a characteristic geographical distribution. They show predominance in Asian and Latin American countries, including Mexico [7-10], and rarely appear in Caucasian countries. This gives them a distinctive racial pattern [11,12]. This type of geographical distribution is closely associated to a high incidence of infection by EBV, as has been extensively reported [1,9,12,13].

In addition to the high positivity of neoplastic cells to the immunophenotype of NK (CD 56) cells and cytoplasmic CD3, the lack of rearrangement of the T-clonal receptor cells and positivity to CD 56 strongly suggest that this type of lymphoma derives from T/NK cells [6,14-16] This particular type of lymphoma is different from other lymphoproliferative varieties by its characteristic destruction of blood vessels, and the progressive necrosis of soft and bone tissues. These changes have been associated to angioinvasion and lysis of the target cells, by the release of cytotoxic granules such as perforins and granzymes present in NK cells and in cytotoxic T lymphocytes [16-18].

Even if the biological information on lymphomas is growing, the invasive capacity and cell destruction of this neoplasm probably due to the participation of proteolytic enzymes, such as metalloproteases has been scarcely explored in head and neck carcinomas and non-Hodgkin's lymphomas and reactive lymphocytes and peritumoral stroma [19-22]. In cancer, in spite of the classical proteolysis of the basal membrane and the extracellular matrix, the different MMPs have been involved in other paths, as the formation of a microenviromment for the transformation of promoters, mediators in the activation of growth factors, apoptosis suppression, destruction of quimocinas and liberation of angiogenic factors. Matrix metalloproteinases are synthesized as inactive zymogens, which are then activated predominantly pericellularly either by other MMPs or by serine proteases. The activity of MMPs is specifically inhibited by the so-called tissue inhibitors of metalloproteases (tisullar inhibitors (TIMPs)). Currently, four different TIMPs are known to exist: TIMPs 1, 2, 3, and 4. Moreover, the particular case of T/NK cell lymphomas has been scarcely explored due to the infrequency of the disease and the difficulty to obtain representative material due to the extensive necrosis. For this reason our objective is to explore the expression of different MMPs in the cells and stroma which are around of the blood vessels damaged and their possible correlation with some clinico-pathological parameters.

## Methods

**Figure 1:**
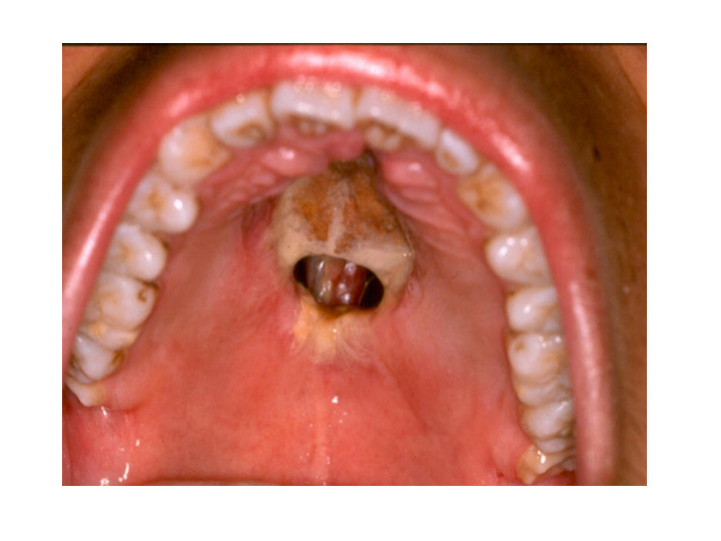
Clinical aspect in patient with T/NK cells angiocentric lymphoma, which shows destructive ulcerative lesion in hard palate and extensive zones of necrosis.

**Figure 2:**
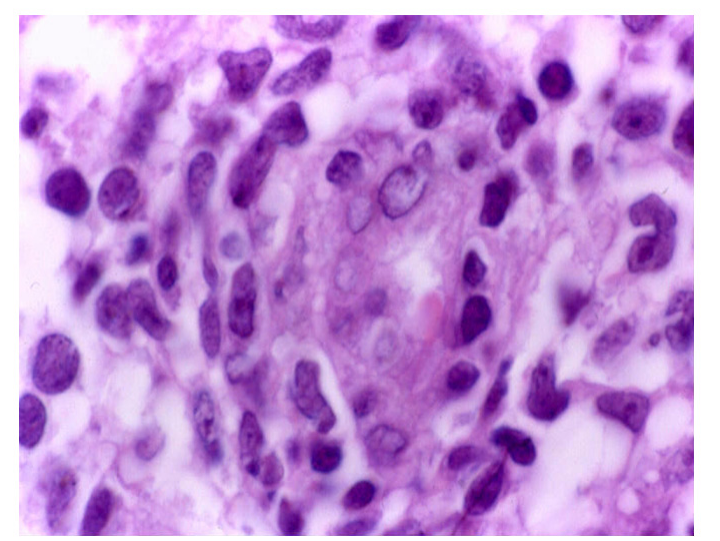
**Histological aspect showing polymorphous cell population with malignant lymphoid cells.** This picture shows prominence of endothelial cells as well as invasion of small and large cleaved neoplastic cells (HE, 400×)

From 31 cases previously studied, 20 nasal type lymphomas were identified as T/NK cells EBV positive from National Cancer Institute of Mexico and used for this study. From this series, 13 were men and seven were women (M:F range 1.8:1) with a median age of 43 (range form 22 to 93 years). In 13 cases (65%) the primary tumor was localized in the nasal cavity, in four patients it was localized in the palate and in three in the nasopharynx (Fig. 1). In 12 patients the treatment was chemotherapy followed by radiotherapy; four patients received chemotherapy only; in three it was only radiotherapy and one patient died before any treatment scheme could be started. Nine patients (45%) presented early disease (clinical stages I and II) and eleven patients (55%), advanced stages (III and IV). Fourteen patients died from the disease (70%); six patients are alive, one with tumoral activity and five of them without it (Table 1). Histopathologically, all cases showed atypical lymphoid cells with angiocentricity and angiodestruction (Fig. 2). In addition, focal or confluent coagulative necrosis was observed in all cases. The morphological spectrum of the atypical lymphoid cells varied from case to case; most cases showed a mixture of medium and large-sized cells (17 cases, 85%) (Table 1). The inflammatory spectrum frequently included plasmocytes, histiocytes, neutrophils and eosinophils. These cells were localized between the tumor cell nests. Three cases showed predominance of large cells with vesicleladen nuclei, apparent nucleoli and frequent mitoses; in these, the inflammatory component was less obvious.

**Table 1:** Clinicopathological findings from 20 patients with extra nodal nasal-type T/NK lymphoma.

**Case**	**Age**	**Sex**	**Tumour localized**	**Histology**	**CD56**	**Clinical stage**	**Treatment**	**Follow-up**
1	57	M	Palate	Mixture	+	III-B	Ct+Rt	DWD (10 m)
2	30	F	Nasal cavity	Mixture	+	I-B	Ct+Rt	DWD (16 m)
3	42	F	Nasal cavity	Mixture	+	IV-B	Ct	DWD (3 m)
4	32	M	Palate	Mixture	+	II-A	Ct+Rt	DWD (8 m)
5	93	F	Nasal cavity	Large cells	+	II-B	Rt	DWD (1 m)
6	49	M	Nasal cavity	Mixture	+	II-B	Ct+Rt	AWOD (16 m)
7	38	M	Nasopharynx	Mixture	+	I-A	Ct+Rt	AWOD (10 m)
8	23	F	Palate	Mixture	+	II-B	Ct+Rt	AWOD (12 m)
9	23	M	Nasopharynx	Mixture	+	III-A	Ct+Rt	AWOD (60 m)
10	28	F	Nasal cavity	Mixture	+	III-B	Ct+Rt	DWD (4 m)
11	67	F	Nasopharynx	Mixture	+	III-B	Ct+Rt	DWD (36 m)
12	43	M	Nasal cavity	Mixture	+	IV-B	Ct	DWD (1 m)
13	62	M	Nasal cavity	Mixture	+	II-B	Rt	DWD (1 m)
14	66	M	Nasal cavity	Large cells	+	II-A	Ct+Rt	AWOD (60 m)
15	36	M	Nasal cavity	Mixture	+	IV-B	Ct	DWD (1 m)
16	54	M	Nasal cavity	Mixture	+	IV-B	Ct+Rt	DWD (3 m)
17	57	F	Nasal cavity	Large cells	+	IV-B	Rt	DWD (1 m)
18	63	M	Nasal cavity	Mixture	+	I-A	Ct+Rt	AWD (132 m)
19	22	M	Nasal cavity	Mixture	+	IV-B	None	DWD (1 m)
20	43	M	Palate	Mixture	+	IV-B	Ct	DWD (3 m)

Ct, chemotherapy; Rt, radiotherapy; DWD, death with disease; AWOD, alive without disease; AWD, alive with disease.

### Immunohistochemistry

Immunohistochemical studies were performed as follows: immunostaining was conducted using an autostainer (Dako Corp) according to the company's protocol. After tissue deparaffinization and slide rehydration, the antigen retrieval was achieved by boiling the preparations in a microwave oven with a 0.001 mol/L of citrate buffer, pH 6.0, containing 0.1% Tween 20, by 30 min. The antibody panel included MMP-1, MMP-2, MMP-3, MMP-9, and two metalloprotease inhibitors, TIMP-1 and TIMP-2 from Oncogene Research Products (Boston, MA, USA); MMP-11 and MMP-13 from Neomarkers, Inc. (Fremont, California, USA). They were used following the manufacturer's recommended protocol for the specific monoclonal antibodies. Primary antibodies were diluted at a concentration of 1:50 and incubated 55 min. A secondary universal biotin-labeled antibody was used. Later it was counter dyed with HE, in order to differentiate the neoplastic versus reactive lymphocytes. The immunostainings were evaluated in tumor, stromal, endothelium and residual epithelial cells (surface and mucus gland ductal epithelium). For evaluations a double-headed microscope was used with a high resolution objective (40×). Percentage of cellular positive to metalloproteases (range from 0 to 100%) and intensity (0, +, ++, +++) were quantified for the IHC studies. Expression was evaluated in neoplastic, endothelial, stromal and residual epithelial cells. When immunohistochemical expression was either absent or weak (0, +) were considered negative. Immunohistochemical expression of ++ or +++ was considered positive.

### Statistical analysis

Statistical analysis was based on chi square and Fisher's exact test and test of Mc Nemar to assess MMP-1, MMP-2, MMP-3, MMP-9, MMP-11, MMP-13, TIMP-1 y TIMP-2 expression of neoplastic cells, peritumoral fibroblasts, endothelium cells and epithelium and its relation to clinico-pathological parameters, using the Sigma Stat Ver. 3.00 software (SPSS, USA). A *p *value < 0.05 was considered statistically significant. The study received an ethical waiver from National Institute of Cancer, Mexico.

## Results

Expression of MMPs varied from moderate to intense in the positive cases. The expression of metalloproteases 1, 2, 3, 9, 11, 13 and TIMP-1 and TIMP-2 and their distribution in tissue is shown in table 2.

**Table 2:** Relationship between expression of the differents MMPs and TIMPs in the cells.

Factor	EvenT frecuency	Tes Mc Nemar ≠ Degree of ASSOCIATION
***MMP-1***		
Epithelium (-) vs (+)	9/11	*P *= 0.004 tumor ≠ epithelium
Tumor (-) vs (+)	0/20	*P *= 0.000 tumor ≠ endothelium
Stroma (-) vs (+)	3/17	
Endothelium (-) vs (+)	18/2	

***MMP-2***		
Epithelium (-) vs (+)	9/11	*P *= 0.031 tumor ≠ stroma
Tumor (-) vs (+)	7/13	*P *= 0.001 tumor ≠ endothelium
Stroma (-) vs (+)	13/7	
Endothelium (-) vs (+)	18/2	

***MMP-3***		
Epithelium (-) vs (+)	11/9	*P *= 0.004 tumor ≠ endothelium
Tumor (-) vs (+)	10/10	
Stroma (-) vs (+)	11/9	
Endothelium (-) vs (+)	19/1	

***MMP-9***		
Epithelium (-) vs (+)	17/3	*P *= 0.031 tumor ≠ endothelium
Tumor (-) vs (+)	14/6	
Stroma (-) vs (+)	16/4	
Endothelium (-) vs (+)	20/0	

***MMP-11***		
Epithelium (-) vs (+)	8/12	*P *= 0.039 tumor ≠ stroma
Tumor (-) vs (+)	1/19	*P *= 0.039 tumor ≠ epithelium
Stroma (-) vs (+)	8/12	*P *= 0.000 tumor ≠ endothelium
Endothelium (-) vs (+)	16/4	

***MMP-13***		
Epithelium (-) vs (+)	12/8	*P *= 0.031 tumor ≠ endothelium
Tumor (-) vs (+)	14/6	
Stroma (-) vs (+)	14/6	
Endothelium (-) vs (+)	20/0	

***TIMP-1***		
Epithelium (-) vs (+)	8/12	*P *= 0.002 tumor ≠ stroma
Tumor (-) vs (+)	19/1	*P *= 0.039 tumor ≠ epithelium
Stroma (-) vs (+)	11/9	*P *= 0.000 tumor ≠ endothelium
Endothelium (-) vs (+)	16/4	

***TIMP-2***		
Epithelium (-) vs (+)	17/3	
Tumor (-) vs (+)	18/2	
Stroma (-) vs (+)	16/4	
Endothelium (-) vs (+)	20/0	

***MMP-2 vs TIMP-2***(+) vs (+)	13/2	*P *= 0.007
***MMP-9 vs TIMP-1***(+) vs (+)	6/1	*P *= 0.001

The expression in all the cases of MMP-1 thus neoplastic, fibroblasts and endothelial cells, may indicate a link action in the degradation of the stroma. The expression of MMP-2 was present basically in the neoplastic cells. In the case of MMP-9 most of the cases were negative both tumoral and reactive cells. The expression of MMP-11 was seen in the neoplastic cells and fibroblasts, but statistically the difference in proportions was significant. Regarding the expression of inhibitors, TIMP-1 was found statistically significant difference regarding the proportion of cases that expressed MMP-9, likewise observed for TIMP-2 regarding MMP-2 (table 2 and 3).

**Table 3:** The cells of tumor, stroma, endothelium and epithelium are 50% positive MMPs and TIMPs in each case.

**Case**	**(+)**	**Tumor**	**Stroma**	**Endothelium**	**Epithelium**
1	***MMP*** ***TIMP***	-1,-2, -11	-2		-1,-2,-3,-11,-13 -1
2	***MMP*** ***TIMP***	-1,-9,-11		-11	-2,-3,-11,-13, -1
3	***MMP*** ***TIMP***	-1,-11	-1,-9,-11,-13 -1,-2	-1	-2,-3,-11,-13,
4	***MMP*** ***TIMP***	-1,-11 -2	-3,-9,-11		-1,-2,-11
5	***MMP*** ***TIMP***	-1,-2,-3,-9,-11 -1	-1,-2,-3 -2		-1,-2,-11,-13, -1
6	***MMP*** ***TIMP***	-1,-3,-11	-1,-11 -1	-1	-1
7	***MMP*** ***TIMP***	-1,-2,-3,-11,-13	-1,-2,-3,-11,-13	-2,-3,-11 -1	-1,-2,-3,-11,-13, -1
8	***MMP*** ***TIMP***	-1,-2,-3,-11,-13	-1,-13		-1,-9 -1,-2
9	***MMP*** ***TIMP***	-1,-2,-3, -11	-1,-2,-3,-11,-13 -1	-1	-1,-3,-11 -1
10	***MMP*** ***TIMP***	-1,-2,-3,-9,-11,-13	-1,-2,-3	-1,-2	-1,-2,-3
11	***MMP*** ***TIMP***	-1,-11	-1,-3,-11	-11	-1,-3,-11
12	***MMP*** ***TIMP***	-1,-2,-3,-11 -2	-1,-3		-2,-11,-13 -1
13	***MMP*** ***TIMP***	-1,-2,-3,-9,-11	-1,-2,-3,-13 -1		-1 -1
14	***MMP*** ***TIMP***	-1,-2	-1,-3,-9 -2		
15	***MMP*** ***TIMP***	-1,-2	-1,-11 -1	-11	
16	***MMP*** ***TIMP***	-1	-1 -1		
17	***MMP*** ***TIMP***	-1	-1 -1		-1,-2,-3,-11,-13 -1
18	***MMP*** ***TIMP***	-1,-2	-1,-9,-11 -2		-2,-11,-13 -1
19	***MMP*** ***TIMP***	-1,-2	-1,-2,-11,-13 -1		-2,-9,-11 -1,-2
20	***MMP*** ***TIMP***	-1,-2	-1,-11 -1	-1	-3,-9 -1,-2

**Figure 3:**
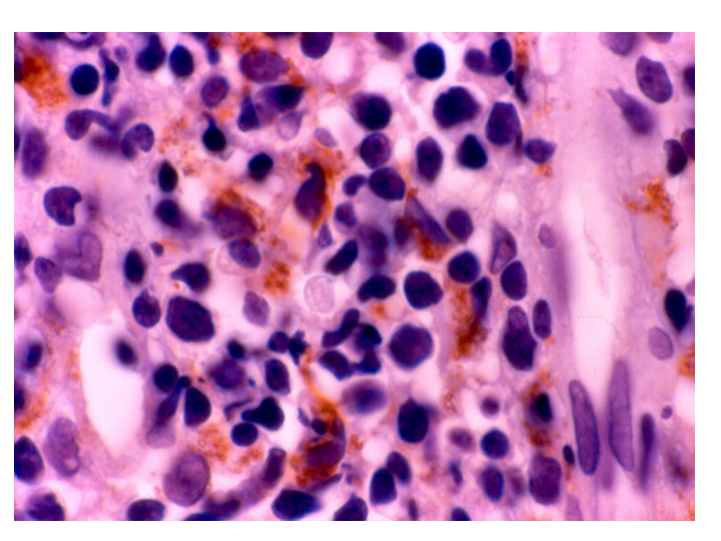
Inmunohistochemical study shows cytoplasmic staining to MMP-11 in neoplastic cells around the blood vessel.

**Figure 4:**
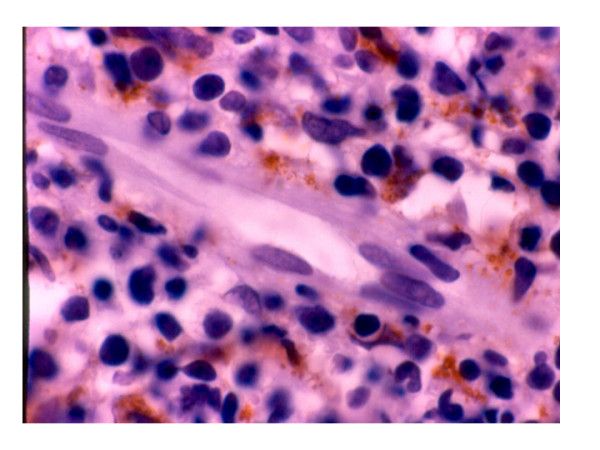
Microscopic image showing expression to MMP-1 in the extracelullar tissue, near of the blood vessel (avidin-biotin complex method).

The MMP-2, -3 and -9 were expressed in neoplastic cells between 30 to 65% of the cases. TIMP-1 was presented mainly in the epithelium and TIMP-2 was poor expresses in most of the cells and cases (Figs. 3 and 4). There were no statistical significance between the different enzymes used and the clinical pathological parameters included, besides status and survival of the patients.

**Figure 5:**
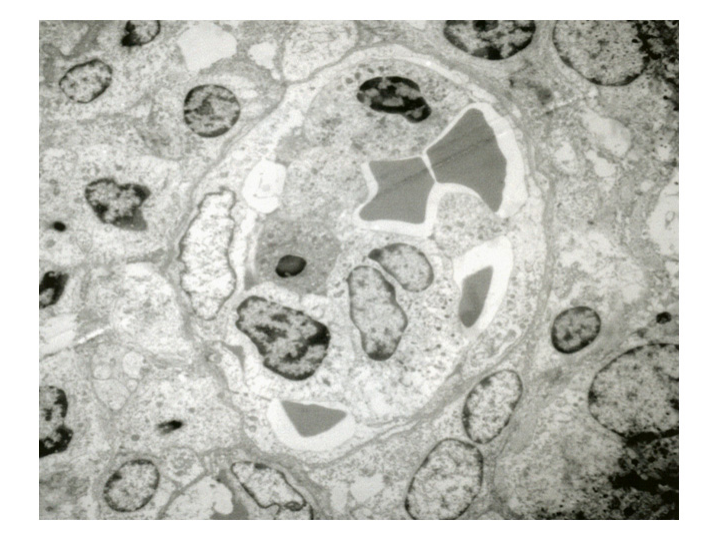
Ultrastructure of capillary, observing neoplastic cell with granules in the cytoplasm in the upper area (11 000 A).

**Figure 6:**
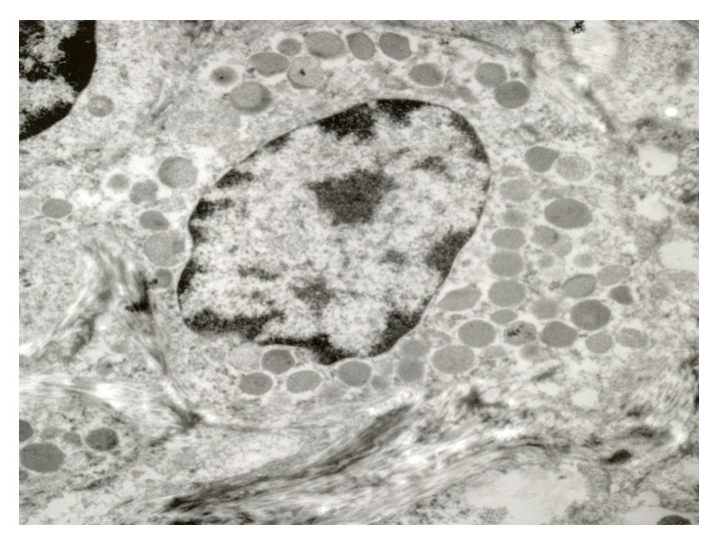
**Closer of the previous image, showing fibres of fragmented collagen with intracytoplasm and extracellular granules.** (13 000 A).

From all the studied cases, two of them were analyzed ultrastructurally, showed prominence of endothelial cells and infiltration of lymphoid-like cells in capillary vessels. These cells showed fissures in the nuclear outline and electrodense granules in the cytoplasm. Some granules were extracellularly located and in contact with subendothelial collagen fibers (Figs. 5 and 6).

## Discussion

The nasal cavity, the palate and in general the midfacial line are regions continuously stimulated by many extraneous antigens. This stimulus creates a permanent and constant interaction between antigens and cells that participate in the host's defense. Among these antigens, a highly antigenic is the EBV. Chronic exposure to this virus can damage NK cells and T lymphocytes, these cells eventually can be transformed with time and generate extranodal lymphomas as T/NK nasal type lymphomas [16,23].

Once a neoplastic lesion is established in the nasal cavity and/or midfacial region, individual tissue modifications occur in the host. This is histologically translated to inflammatory infiltrate of macrophages, granulocytes, lymphoid and plasmatic cells. This reactive tissue response can mask the disease and make the diagnosis of lymphoma more difficult [3,7,10].

The chronic exposure of the nasal and palatine mucosa to EBV is probably added to a genetic and racial predisposition, which can explain the predominant geographic distribution of the disease in some countries of Asia and Latin America, including Mexico, a country which shows an increasing number of patients with this malignancy [7-12]. These two factors, EBV and the host's tissue response can lead to the induction of enzymatic processes that destroy the extracellular matrix of the blood vessel wall and thus generate progressive areas of necrosis of soft and bone tissue.

A previous study performed in Mexico in 23 cases of T/NK lymphoma showed that 96% of the cases exhibited expression of cytotoxic granules of TIA-1 and perforins inside neoplastic cells [16]. Eighteen of these 23 cases were included in this study.

In addition to the immunohistochemistry results, two of the cases in the present series were analyzed with electron microscopy; electrodense granules were observed in the cytoplasm of the neoplastic cells. Besides, these granules were identified in the blood vessel wall and were observed disaggregating subendothelial collagen fibrils, which strongly suggests a destructive action of the vascular wall that contributes to angiodestruction. These findings have also been observed and published by other authors [23,24].

The phenomena of angiodestruction and necrosis could be multifactorial and, in addition to the mentioned mechanisms, could be potentiated by the action of proteolytic enzymes such as metalloproteases. These MMPs are a group of Zn dependent endopeptidases, which break down a large variety of molecules among them fibronectin, laminin, vitronectin, type IV collagen, thrombospondin, elastin, hyaluronic acid, factor VIII, heparan sulfate, proteoglycans, among others [25]. In addition, they can activate, and in turn be activated by growth factors, and thus promote degradation, migration, differentiation and invasion processes [21,26].

The tumor or stromal expression of these enzymes has been associated to a more aggressive behavior of malignant neoplasms; in particular, they have been studied in head and neck, and colon carcinoma, among others, and their presence is associated to a poor prognosis [22,26-28]. In this series, the expression of MMP-1 both in tumor and in peritumoral fibroblasts, and of MMP-11 in neoplastic cells, could explain the phenomenon of break-down of cell elements related to the blood vessel wall, such as type IV collagen, laminin and fibronectin. It has been shown that MMP-1 is actively secreted by tumor cells. This immunohistochemical study confirms the phenotypic expression of MMP-1 in tumor cells and shows that its intensity increases as the tumor cells come in closer contact with the vascular wall.

There, MMP-1 could be contributing to the degradation of subendothelial collagen and, with it, participate in the degradation of the blood vessel wall. Some studies show that the cells of non-Hodgkin lymphoma destroy the extracellular matrix through the intense expression of MMPs. Those more intensely expressed are MMP-9 and TIMP-1, and their presence has been associated with a poor prognosis [20,21]. In our results the percentage of cases which expressed MMP-9 and TIMP-1 was minor (Table 2). The TIMPs are involved in complicated biological functions as cellular morphologic changes, stimulation for the growth of different cellular types and inhibition of angiogenesis among others. TIMP-1 and 2 were identified originally like inhibiting of MMP-9 and MMP-2 respectively. TIMP-2 is a discreet regulator in the activation of MMP-2, for a ternary complex with proMMP-2 and MT1-MMP [19,22]. In this regard, it is interesting to note that in some lymphoma cell lines, MMP-9 is induced by the EBV latent membrane protein-1 (LMP-1), particularly in Burkitt's lymphoma [29]. In experimental studies of cultured tumor cells infected with EBV that express LMP-1, overexpression of MMP-9 is observed, which is activated through the nuclear factor NFkB pathway.

These same studies performed *in vitro*, show that the use of salicylates decreases the invasive capacity of the tumor cell lines, as well as their MMP-9 secretion by blocking the NFkB signaling pathway [30]. In the present study, MMP-9 was not intensely expressed, probably because in this type of T/NK cell extranodal lymphoma lack of participation of the adequate stromal elements such as fibroblasts, which are more evident in carcinomas.

MMP-2 expression in the neoplastic cells of 13 cases of this series can be due to MMP-1 and MMP-11 activation, which enter an enzymatic cascade that activates the expression of MMP-2, thus contributing to the vascular degradation process.

In this study, practically none of the endothelial or tumor cells expressed TIMP-1 or TIMP-2. These findings suggest an imbalance in metalloprotease over inhibitor production, and thus, the relative ease with which neoplastic cells and their chemical components break down the molecular and cellular components of the vascular wall. On the other hand, 12 cases of the present series showed a more intense expression of TIMP-1 in epithelial cells, mainly the mucosal gland, whose structures showed scarce morphological damage, even when the ducts were immersed in tumor cell zones. It may be that the epithelial cells of the mucosal glands secrete more MMP inhibitors at least during a disease period. Endothelial cells showed no statistically significant expression of MMP or TIMP in comparison with neoplastic, epithelial and stroma cells; these endothelial cells only seem to be a target of cell damage, at least in this disease. It is possible that the non statistical significance in this series is due to the size of the number of cases studied.

Aoudjit et al, showed the induction of some MMPs in T cell lymphoma in contact with endothelial cells through the interaction of intercellular adhesion molecules (1/LFA-1 molecule) [31]. This same type of mechanism could be occurring between endothelial and neoplastic cells, and MMP-1 and MMP-11. Non-Hodgkin lymphomas are a large and heterogeneous group of tumors, which differ, in biological aggressiveness and clinical course. Of them, the nasal type extranodal T/NK cell lymphomas constitute a group of highly biological aggressiveness and patients normally have limited therapeutic options and a fatal prognosis in the short term [32].

Although the series presented here are small, it is possible that directed treatment to inhibit the action of specific metalloproteases present in neoplastic cells can be a therapeutic alternative. Also, inhibition of cytotoxic granule production by NK cells should be attempted, and the use of salicylates should be evaluated in lymphomas exposed to EBV to inhibit the NFkB signaling pathway [30,33].

## Conclusion

Nasal-type extranodal T/NK cell lymphomas, characterized for invasion, destruction of vascular walls, and extracellular matrix. This damage is cause by proteolytic enzymes, and particularly in this disease by metalloproteases MMP-1 and MMP-11, whose mechanism is probably related to the participation of the Epstein-Barr virus. It is necessary to study more enzymes and focus them to quantify and determine their activity, in order to have a better correlation with histological features in this type of neoplasm. The present study showed that the expression of MMP-2 and MMP-9 was not significant, as shown in other neoplasms, particularly in carcinomas, whose difference with this type of lymphoma resides in that stromal cells such as fibroblasts are the main component cell involved in the epithelial malignant tumors.

In addition, the use of synthetic agents that produce TIMPs and factors that participate in the inhibition of cytotoxic granule secretion could be a therapeutic alternative. The activity of the TIMPS have been poor conclusive, because of the dual participation and because the over expression in the different kind of tumors, for that reason their utility as treatment is not acceptable yet.

## Abbreviations

T/NK: T/natural killer; EBV: Epstein Barr Virus; MMPs: Metalloproteases; TIMPs: Tissue inhibitors of metalloproteases.

## Competing interests

The authors declare that they have no competing interests.

## Authors' contributions

AM realized the selection cases, design of this study and wrote the manuscript. AMB checked the text of the manuscript and realized the statistical analysis. JHA did the analysis and revision of the methodology and discussion. ABM searched the bibliography and edited the manuscript. LSR did the evaluation of immununohistochemestry and analysis of the results. LRG participated in the cases selection, photo material and she did the correction suggested by the reviewers. She is the author correspondence of this manuscript. All authors read and approved the final manuscript.

## References

[B1] Jaffe ES, Chan JKC, Su I-J, Frizzera G, Mori S, Feller AC, Ho FCS (1996). Report of the workshop on nasal an related extranodal angiocentric T/natural killer cell lymphomas. Definitions, differential diagnosis, and epidemiology. Am J Surg Pathol.

[B2] Campo E, Cardesa A, Alos L, Palacin A, Cotarro J, Traserra J, Montserrat E (1991). Non-Hodgkin's lymphomas of the nasal cavity and paranasal sinuses, and immunohistochemical study. Am J Clin Pathol.

[B3] Gaulard P, Henni T, Marolleau JP, Haioun C, Henni Z, Voisin MC, Divine M, Goossens M, Farcet JP, Reyes F (1988). Lethal midline granuloma (polymorphic reticulosis) and lymphomatoid granulomatosis: evidence for monoclonal T-cell lymphoproliferative disorder. Cancer.

[B4] Eichel BS, Harrison EG, Devine KD, Scanlon PW, Brown HA (1996). Primary lymphoma of the nose including a relationship of lethal midline granuloma. Am J Surg.

[B5] Jaffe ES (1984). Pathologic and clinical spectrum of post-thymic T-cell malignancies. Cancer Investigation.

[B6] Nakamura S, Suchi T, Koshikawa T, Kitoh K, Koike K, Komatsu H, Iida S, Kagami Y, Ogura M, Katoh E (1995). Clinicopathologic study of CD 56 (NCAM)-positive angiocentric lymphoma occurring in sites other than the upper and lower respiratory tract. Am J Surg Path.

[B7] Aozasa K, Ohsawa M, Tajima K, Sasaki R, Maeda H, Matsunaga T, Friedmann I (1989). Nation-wide study of lethal midline granuloma in japan: frequencies of Wegener's granulomatosis, polymorphic reticulosis, malignant lymphoma and other related conditions. Int J Cancer.

[B8] Ho FCS, Todd D, Loke SL, Ng RP, Khoo RKK (1984). Features of malignant lymphomas in 294 Hong Kong Chinese patients, retrospective study covering an eight year period. Int J Cancer.

[B9] Arber DA, Weiss LM, Albujar PF, Chen YY, Jaffe ES (1993). Nasal lymphomas in Peru, high incidence of T-cell immunophenotype and Epstein-Barr virus infection. Am J Surg Pathol.

[B10] Navarro-Roman L, Zarate Osorno A, Meneses-Garcia A, Kingma DW, Jaffe ES (1994). High grade AIL and Epstein-Barr virus infection in 22 cases from Mexico. Mod Pathol.

[B11] Gaal K, Sun NCJ, Hernandez AM, Farber DA (2000). Sinonasal T/NK cell lymphomas in the United States. Am J Surg Pathol.

[B12] Ng SB, Lai KW, Murugaya S, Lee KM, Loong SL, Fook-Chong S, Tao M, Sng I (2004). Nasaltype extranodal natural killer/Tcell lymphomas: a clinicopathologic and genotypic study of 42 cases in Singapore. Mod Pathol.

[B13] Khania F, Yao Q-Y, Niedobitek G, Sihota S, Rickinson AB, Young LS (1996). Analysis of Epstein-Barr gene polymorphisms in normal donors and in virus-associated tumors from different geographic locations. Blood.

[B14] Chan JK, Sin VC, Wong KF, Ng CS, Tsang WY, Chan CH, Cheung MM, Lau WH (1997). Non-nasal lymphomas expressing the natural killer cell marker CD 56: a clinicopathologic study of 49 cases of an uncommon aggressive neoplasm. Blood.

[B15] Quintanilla-Martinez L, Jaffe ES (2000). Aggressive NK cell lymphoma insights in to the spectrum of NK cell derived malignancies. Histopathology.

[B16] Elenitoba-Johnnson KJJ, Zarate-Osorno A, Meneses A, Krenaccs L, Kingma DW, Raffeld M, Jaffe ES (1998). Cytotoxic granular protein expression, Epstein-Barr virus strain type, and latent membrane protein-1 oncogene deletions in nasal T-lymphocyte/natural killer cell lymphomas from Mexico. Mod Pathol.

[B17] Tiang Q, Streuli M, Saito H, Schlossman SF, Anderson P (1991). A polyadenylate binding protein localized to the granules of cytolytic induces DNA fragmentation in target cells. Cell.

[B18] Smyth MJ, Trapane JAA (1995). Granzymes: exogenous proteinases that induce target cell apoptosis. Immunol Today.

[B19] Egablad M, Werb Z (2002). New functions for the matrix metalloproteinases in cancer progression. Nature Reviews/Cancer.

[B20] Kossakowska AE, Edwards DR, Prusinkiewicz C, Zhang MC, Guo D, Urbanski SJ, Grogan T, Marquez LA, Janowska-Wieczorek A (1999). Interleukin-6 regulation of matrix metalloproteinase (MMP-2 and MMP-9) and tissue inhibitor of metalloproteinase (TIMP-1) expression in malignant non-Hodgkin's lymphomas. Blood.

[B21] Stetler-Stevenson M, Mansoor A, Lim M, Fukushima P, Kehrl J, Marti G, Ptaszynski K, Wang J, Stetler-Stevenson WG Expression of matrix metalloproteinases and tissue inhibitors of metalloproteinases in reactive and neoplastic lymphoid cells. Blood.

[B22] Franchi A, Santucci M, Masini E, Sardi I, Paglierani M, Gallo O (2002). Expression of matrix metalloproteinase 1, matrix metalloproteinase 2, and metalloproteinase 9 in carcinoma of the head and neck. Correlation with p53 status, inductible nitric oxide synthase activity, and angiogenesis. Cancer.

[B23] Yamanaka N, Nakamura A, Minase T, Sambe S, Ishi Y (1985). Tcell lymphoma: characterization by monoclonal antibodies. Ann Otol Rhinol Laryngol.

[B24] Nava VE, Jaffe ES (2005). The pathology of NK-cell lymphomas and leukemias. Adv Anat Pathol.

[B25] Ioachim EE, Athanassiaadou SE, Kamina S, Carassavoglou K, Agnantis NJ (1998). Matrix metalloproteinase expression in human breast cancer: an immunohistochemical study including correlation with cathepsin D, type IV, collagen, laminin, fibronectin, EFGR, c-erbB2 ocoprotein, p53, steroid receptors status and proliferative activity. Anticancer Res.

[B26] Kurahara S, Shinohara M, Ikebe T, Nakamura S, Beppu M, Hiraki A, Takeuchi H, Shirasuna K (1999). Expression of MMPs, MT-MMP, and TIMPs in squamous cell carcinoma of the oral cavity: correlations with tumor invasion and metastasis. Head Neck.

[B27] Harada T, Shinohara M, Nakamura S, Oka M (1994). An immunohistochemical study of extracellular matrix in oral squamous cell carcinoma and its association with invasive and metastatic potential. Virchows Arch.

[B28] Zeng ZC, Cohen AM, Zhang ZF, Stetler-Stevenson W, Guillem JG (1995). Elevated tissue inhibitor of metalloproteinase 1 RNA in colorectal cancer stroma correlates with lymph nodes and distant metastasis. Clin Cancer Res.

[B29] Yoshizaki T, Sato H, Furukawa M, Pagano JS (1998). The expression of matrix metalloproteinase 9 is enhanced by Epstein-Barr virus latent membrane protein1. Proc Natl Acad Sci USA.

[B30] Murono S, Yoshizaki T, Sato H, Takeshita H, Furukawa M, Pagano JS (2000). Aspirin inhibitors tumor cell invasiveness induced by Epstein-Barr virus latent membrane protein 1 through suppression of matrix metalloproteinase-9 expression. Cancer Res.

[B31] Aoudjit F, Potworowski EF, St-Pierre Y (1998). bi-directional induction of matrix metalloproteinase-9 and tissue inhibitor of matrix metalloproteinase-1 during T-lymphoma/endothelial cell contact. J Immunol.

[B32] Quintanilla-Martinez L, Kremer M, Keller G, Nathrath M, Gamboa-Dominguez A, Meneses A, Luna-Contreras L, Cabras A, Hoefler H, Mohar A, Fend F (2001). p53 mutations in nasal natural killer/cell lymphoma from Mexico. Association with large cell morphology and advanced disease. Am J Pathol.

[B33] Folgueras AR, Pendas AM, Sánchez LM, López-Otín C (2004). Matriz metalloproteinases in cancer: from new functions to improved inhibition strategies. Int J Dev Biol.

